# A generalized classification and coding system of Human Disease Animal Model Resource data with a case study to show improving database retrieval efficiency

**DOI:** 10.1371/journal.pone.0281383

**Published:** 2023-02-09

**Authors:** Huiping Li, Wenjuan Zhang

**Affiliations:** 1 Guangdong Laboratory Animals Monitoring Institute, Guangdong Key Laboratory of Laboratory Animals, Guangzhou, Guangdong, China; 2 School of Information Science and Technology, School of Cyber Security, Guangdong University of Foreign Studies, Guangzhou Guangdong, China; China University of Mining and Technology, CHINA

## Abstract

**Background:**

Currently there is no unified data classification and coding standard for the existing human disease animal model resource data worldwide. Different data classification and coding systems produce different retrieval methods. Some of these methods are inefficient and difficult to use. This research investigated the rules for the classification and coding of such data based on the *Replication Methodology of Animal Models for Human Disease*, the Classification and Coding Rules for Health Information Data Set (WS/T 306–2009), the Science and Technology Resource Identification (GB/T 32843–2016), the Scientific Data Management Measures (000014349/2018-00052), and *The Generic Description Specification for Natural Science and Technology Resources*. This research aimed to develop a classification and coding system for data obtained from human disease animal model resource based on the Internet environment to provide a standardized and unified foundation for the collection, saving, retrieval, and sharing of data from this resource.

**Results:**

A complete data classification and coding table compiled in the form of letters and numbers was produced, with a classification infrastructure that expanded layer by layer according to the three dimensions (namely, system diseases, animal species, and modeling methods) and essential attributes. When necessary, it adopted the hierarchy of major, intermediate, and minor categories for certain layer and also one-to-one matched the code and classification result.

**Conclusion:**

Through this study, a sharing and joint construction mechanism for data from this resource can be developed by all research institutes in this field. As a case study, this research also offered technical support for constructing the database for the National Human Disease Animal Model Resource Center. The technological innovation of this paper is to derive a research oriented retrieval method, which provides technical support for the research on the current COVID-19 epidemic and on possible future epidemics.

## Introduction

Human disease animal models (hereinafter referred to as animal models) comprise a laboratory animal with simulated human disease and related material and are established models used in the biomedical field. Animal models are substitutes for studying human disease [1]. Human disease animal model resources (hereinafter referred to as animal model resources) are not only vital supports for life science research and the biomedicine and other industries but also a strategic resource for science and technology [2]. Researchers working for the prevention and control of corona virus disease 2019 (COVID-19) generally believe that the five main aspects of clinical treatment and medicine, vaccine development, detection technology and products, viral pathogens and epidemiology, and animal model construction will provide a strong technological support. Statistics indicate that to date, >5,000 valuable animal models have been developed worldwide, and nearly 1,000 of these are developed in China [1]. According to *The Laboratory Animal Resource Survey and Development Trend in China* [3], 791 new types of animal models have been developed in China between early 2013 and the end of 2015, including models for diabetes, hypertension, hyperlipidemia, tumor, and inflammation. However, research on data management, data analysis, and data mining of animal model resources is currently lacking in China. The animal model resources developed by different Chinese research institutions are scattered, and the animal model resource databases are established separately. Moreover, the data structures in each database are not unified and the technical standards are inconsistent; thus, all resource data cannot be openly shared. Such poor data management and exchange results in inefficient data sharing of animal model resources.

The COVID-19 epidemic has had a profound impact on our world in both natural and social aspects. From a natural viewpoint, a large number of studies [4] have revealed that not only COVID-19 but also the spatiotemporal dynamics of other similar pathogenic viruses and microorganisms in their corresponding environmental divisions are profoundly changing our living environment. Facing this challenge, it is widely believed technology innovation is the necessary means to solve this urgent new problem.

Technological innovation is an important driving force for the long-term development of human society. In today’s society, it is also a vital means for enterprises to gain competitive advantage. In recent years, research on the source, diffusion, and upgrading of technological innovation has received much attention [5]. The research found that both radical and incremental innovation problems are indispensible preconditions and reasons for triggering enterprises and research institutions to carry out technological innovation [6].

From a social perspective, the world’s political, economic, medical and health environment has undergone profound changes. Controlling the spread of such an epidemic is a new problem facing governments all over the world. So, are all countries ready to face this sudden challenge? The latest research shows that the government’s preparedness is worrying [7].

Government governance is critical to the process and outcome of the epidemic. Studies have shown that the stronger the governance capacity of the government, the higher the vaccination rate of the country [8]. In the face of the current situation of many infected people in many countries in the process of epidemic prevention, the researchers tried to help the government design strategies to support various policy responses and provide decision-making ideas for other types of infectious disease threats that may occur in the future [9].

Animal models are one of the pillars for the study of COVID-19 disease, which is caused by severe acute respiratory syndrome corona virus 2 (SARS-CoV-2). The research results of animal models have a subversive impact on the past universal understanding of this virus. In the past, scholars generally believed that this virus infected human testes through angiotensin-converting enzyme 2 (ACE2). Through the study of the conventional C57BL/6 mouse model, people found that the past understanding may be wrong. The SARS-CoV-2 may infect human testes through Sertoli cells rather than ACE2 [10]. In addition to helping to discover the pathogenesis, animal models have also made great contributions to the development of medicines for treating human diseases. A case in point here is the cardiovascular drug is developed from a monkey model [11]. Similarly, researchers believe animal models are of positive significance for the development of COVID-19 clinical medicines.

In the information era, the data-based expression of animal models and the resulting database management of animal model resources have more and more important scientific significance. However, in some cases, it is very difficult to realize the data-based expression of animal models. A study on common marmoset facial action coding system shows that researchers have made great efforts to adopt objective measurement technology and avoid subjective interference in the process of animal facial expression coding [12]. Metabolomics is a powerful tool for developing traditional drugs. However, it is difficult to classify metabolomic data because of its high throughput, scarcity, high dimension, and small sample size. The researchers improved a stacked automatic encoder to complete data classification and establish relevant Naru3 (a traditional Mongolian medicine) animal models [13].

In the face of such difficulties, fortunately, the development of data processing technology itself has provided a powerful means for data management of animal models. Considering the characteristics of data such as multi-source, heterogeneity and imbalance, embedded multi-function data mining technology based on granular computing has been developed [14]. In order to use time series health data sets for selective prediction, researchers developed an algorithm using long short-term memory and unit-wise batch standardization [15]. Due to the lack of standard analytical research methods in the field of psychology, there has been a long-standing problem of replication research difficulty. Recently, researchers have adopted new two-layer feedforward neural network architecture to solve the problem of behavioral data classification and psychological data classification, thus replicating the research in this field [16]. According to the past research on stroke for more than 10 years, the data transparency and sharing in the research process is very important. Improving data standardization and data quality management is a prerequisite for achieving data transparency and sharing [17]. With the development of data processing technology described above, more and more animal model information has been data-based expressed successfully. The establishment of animal model resource database is a matter of course.

In a broad sense, the laboratory animal database includes the animal model database. Strictly speaking, there are differences between them. They are different types of databases. Laboratory animal database is a professional database that stores biological characteristic data of laboratory animals and realizes data resource sharing. At present, many laboratory animal resource databases are available worldwide, including the Mouse Genome Informatics of Jackson Laboratory in the USA, the Mutant Mouse Regional Resource Center of the National Institute of Health in the USA, the European Mouse Mutant Archive in Europe, the European Mouse Mutant Cell Repository in Europe, and the Asian Mouse Mutagenesis Resource Association in Asia.

Different from the laboratory animal database, the animal model database is a professional database that stores biological and pathological data of animal with certain human disease, as well as a set of technical methods created, introduced, collected, preserved, supplied the animal model and of animal experiments by researchers, and constantly improves data management and provides data sharing. Compared with the laboratory animal database, the animal model database was built later, with a smaller number and scale. At present, most animal model databases in the world are specialized databases for certain diseases. For example, the MUGEN mouse database in the USA is an animal model database dedicated to human immune disease research; TISMO (Tumor Immune Syngeneic MOuse) of the USA is an animal model database used to research the syngeneic mouse tumors for tumor immunity and immunotherapeutic response.

In March 2020, with support from the Research Institute of Medical Laboratory Animals, Chinese Academy of Medical Sciences, the National Human Disease Animal Model Resource Center, which includes the first national-level comprehensive human disease animal model database in China, was established by the Chinese Ministry of Science and Technology. This resource is classified into the category of experimental materials of Chinese scientific and technological resource in Science and Technology Resource Identification (GB/T 32843–2016) [18] and the identifier is setup according to this specification. However, a normalized and standardized data classification and coding system for relevant animal model resources is currently lacking. The classification and coding system in this research is expected to provide technical support in terms of data classification and coding for the construction and sharing of animal model databases in research units at all levels. With the continuous expansion of animal model resources, a significant amount of data on model classification and essential attributes describing the model characteristics have accumulated; this has led to an increase in the need for scientific and standardized management of such data. To address the limited data-sharing technology for animal model resources, this study explored a set of standardized, dynamic data classification and coding method that had expandability and flexibility. This method can provide technical support for the construction of standardized databases of animal model resources and data co-construction and sharing. It is also conducive to the integration and sharing of animal model resources in China.

In the field of laboratory animal research, researchers have long recognized that laboratory animal databases are vital for research efficiency and implementation of the 3Rs (replacement, refinement, and reduction) [19]. In early research, researchers have paid close attention to the search technology for data on laboratory animals available on the Internet; such technology is useful to make effective use of the database [20]. Some studies have demonstrated how to efficiently find scientific data in appropriate databases such that suitable laboratory animals’ alternatives can be found and the application of the 3Rs can be improved in the field of laboratory animals [21]. Furthermore, other researchers have actively introduced the main contents, functions, and application methods of professional biological database for laboratory animals [22–24].

With expanding research, database construction should be diversified, professionalized, and of high quality. To reduce the number of experiments and lessen the severity of the disease symptoms in animals, some researchers have developed a database for the doses of some active compounds in animals [25]. In recent years, some special databases, such as those of multimodal images and metadata collected in laboratory animal image research, have also been developed [26]. These databases play a special role in research by means of improving the database quality. Some human disorders, such as autism, have their own databases, and the integration of animal models within these special databases may provide an important reference for the treatment of these disorders [27]. Many researchers have investigated the response of laboratory animals to tuberculosis vaccines and established a database for it [28]. With the development of genetic sciences, gene databases for laboratory animals have also been established [29]. Researchers have made several attempts, such as integrating all relevant information, including image caption information, into database to improve database quality and the relevance of data retrieved from the database [30].

China conducts a variety of experiments on laboratory animals and is an important participant in laboratory animal science. Laboratory animal resource databases have been actively constructed in recent years [31]. A series of rules and regulations have been established by the government for database development and application, and the 3Rs are implemented [32]. At present, China’s laboratory animal databases are large scale databases with huge amount data. There are approximately 10 national-level laboratory animal resource databases, including the National Natural Science and Technology Resource Platform Laboratory Animal Resource Center, and the National Genetic Engineering Mouse Resource Center, etc. Moreover, research institutions and biological manufacturing enterprises have created many different level professional laboratory animal databases. With the development of genetic technology, some special genetic engineering databases have also been established [33]. In March 2020, at the time of the COVID-19 pandemic, China announced the establishment of the National Human Disease Animal Model Resource Center [34].

So far, the National Human Disease Animal Model Resource Center has collected 865 existing human disease animal model resources. This shows that the database is still under construction. Research on the classification and coding during database construction process is important to ensure the scientific nature of the database and to enable efficient database retrieval. Currently, data can be retrieved from the National Human Disease Animal Model Resource Center in the form of basic retrieval or advanced retrieval.

Basic retrieval uses two methods. Method 1: use the model identifier, i.e., a unique code of each animal model derived from the unified coding method on the National Science and Technology Infrastructure. The model identifier (for example, CSTR:16397.09.0C01001234, total 21 bits) comprises four parts that successively represent resource code (here is CSTR, total 4 bits, acronym of China Scientific and Technological Resources), registered R&D institutions code (here is 16397, total 5 bits, the code of National Human Disease Animal Model Resource Center), resource type (here is 09, total 2 bits, represent laboratory materials), and unit self-compiled resource code (here is 0C01001234, total 10 bits, it is encoded independently by National Human Disease Animal Model Resource Center.). As model codes are difficult to obtain, basic retrieval of method 1 cannot be used in the practical work. Method 2: use the key words. Key words of animal models mainly include animal name, disease name and other important information about model attributes. For example, model CSTR:16397.09.0C01001234, the English name is SARS-CoV-2 infected transgenic mice model, the key words include SARS-CoV-2, transgenic mice, ACE2, infection. If you use Method 2 to type a keyword in the dialog box, such as transgenic mice, you get a list of 171 model names, and you need to search the list again. So far, the database has not provided the function of refining the search results. Researchers can only use their eyes to browse the model name one by one in the search results list until they find the model they want.

Advanced retrieval uses a simple field-matching method. At present, this national database includes six fields: resource Chinese name, resource English name, disease description, cooperation mode, date, and saving mode.

Because the first three fields comprise many characters, they only need to be partially matched, and the last three fields need to be completely matched. The Boolean logical relationship between fields is *and*. Database users can use one or more fields at a time for retrieve. For example, to retrieval model CSTR:16397.09.0C01001234, a researcher type “SARS-CoV-2” in the resource English name dialog box, and “infection” in the disease description dialog box (This word must use Chinese. It is a part of disease description of this model.). The result of this retrieval is a model name list of 3 models. The rest work for researchers is still to conduct a secondary search by their eyes.

Advanced retrieval improves retrieval efficiency to some extent, but it still has two problems. First, the advanced searcher must know the resource name, disease description, cooperation mode and other specific contents in advance. If all these contents have been obtained, why do researchers need to search this model? Second, the content entered in the dialog box is only a fragment of all characters, but the content of this fragment must be 100% consistent with the retrieved content. Any modification to the retrieved content cannot be accepted by the system.

Moreover, the database does not provide any help with the format for these fields (except the date field). This also makes it difficult to use the advanced retrieval option during actual work.

To improve the quality of database construction, increase the efficiency of database use, and upgrade the level of resource sharing to realize the 3Rs of laboratory animal databases, this study proposes a new data classification and coding system to improve the retrieval efficiency.

## Study design

### Materials and methods

The research sample of this paper is 865 animal models in the National Human Disease Animal Model Resource Center. Each animal model in this center is a specific observation. The research population of this paper is all animal model resources in the world. Due to the difficulty of collection, this paper uses the above mentioned sample to study. From the perspective of sample size, this capacity is suitable for studying database retrieval problems. From the perspective of sample representativeness, it is an appropriate representative of the population and retains the diversity of population. From the perspective of availability, it is an open database that can be retrieved for free.

In the field of information classification and coding [35, 36], the form of information is data. The correct understanding of various information concepts depends on information classification. The unanimous consent of all kinds of information expression depends on information coding. According to the attributes or characteristics of information content, information is classified according to certain principles and methods, and a certain classification system and order are established. Information coding is to endow coding objects with symbols that have certain rules and are easy to be recognized and processed by computers and people, forming a set of code elements. Classification and coding of data in information system is the premise of data processing and communication.

In general, an animal model includes the following information contents: disease category and name, animal category and name, modeling method, and other essential attribute information contained in the model, such as model phenotypic data, judgment index, preservation method, and publications, etc. The classification and coding of animal models are based on the above information contents.

According to the history of animal model research, there are three independent taxonomic criteria for animal models, namely: system disease, animal species and modeling method. If these three taxonomic criteria are combined, the combination is a three dimensions classification system. Let’s take a hypothetical example to see the classification results. If there are 10 major categories of system diseases, 6 major categories of animal species, and 3 modeling methods, the final classification result is that the total animal models can be divided into 180 categories (10*6*3 = 180) at most. Considering the fact that we currently have more than 5,000 animal models, there must be many animal models in each subcategory of our classification results. From the perspective of retrieval efficiency, such classification cannot achieve the efficiency of retrieving most models at one time. A natural idea to improve retrieval efficiency is that we should subdivide each taxonomic criterion at more layers, so that each criterion will be divided into more categories. In this way, our 3 dimensions multi-layer classification system will divide more subcategories.

For system disease, we adopt the taxonomic criterion from the *Replication Methodology of Animal Models for Human Disease*, an authoritative classic book in laboratory animal industry, as the criterion, and carried out three layers (major, intermediate, and minor categories) of hierarchy.

For animal species, we adopt the taxonomic criterion from *The Generic Description Specification for Natural Science and Technology Resources*. This is a normative document of the Chinese Ministry of Science and Technology to describe the generic attributes of science and technology resources and used for the construction of the China Science and Technology Resource Sharing Network. It has the authority from the government. In this study, a three-layer hierarchy is adopted for animal species. The major category refers to laboratory animal only (We keep this layer so that our code can be compatible with higher level databases.); the intermediate category refers to animal species; the minor category refers to animal strain.

For modeling method, the taxonomic criterion from the *Replication Methodology of Animal Models for Human Disease* is applied again. In this research, a two-layer hierarchy is adopted.

So far, a three-dimensional and multi-layer hierarchy of animal model classification framework has been built. According to the preliminary test, this framework can basically ensure that only one or a few animal models are included in each subcategory in the classification results. The success rate of retrieval at one time is greatly improved. However, the number of animal models is not evenly distributed in each subcategory according to our taxonomic criteria. There are still a large number of animal models clustered in some subcategories in the classification results. For example, if we use advanced search in the National Human Disease Animal Model Resource Center database, type mouse in the model name dialog box and type tumor in the disease description dialog box, the result is a 16 pages list of 181 model names. Considering that there are only three categories of modeling methods, and the vast majority of mouse models adopt inducing modeling methods and genetic engineering modeling methods, the tumor mouse inducing model and tumor mouse genetic engineering model in the subcategories separated by the three-dimensional and multi-layer hierarchy classification system will certainly have multiple models clustered. This makes it impossible for researchers to obtain the model they want through retrieval at one time. This occurred in a sample with only 865 observations. Consider the reality that the total number of models is more than 5,000, this clustering phenomenon in a subcategory after classification will be very serious.

Generally speaking, there are two ways to solve this problem. One is through secondary retrieval; the other is to add new taxonomic criterion to make the subcategories of classification results smaller. For the purpose of this research is to improve retrieval efficiency, the second way is better than the first one.

In general, the essential attribute data of each model are recorded as a dataset. The essential attribute data often include: model identifier, model name, disease overview, experimental animal background, modeling method, model phenotype data, evaluation and certification, preservation method, cooperation method, sharing method, relevant literature, etc. The three taxonomic criteria for classifying animal models are the information extracted from essential attribute data that can be classified. In addition, is there any other information that can be classified? The research found that the preservation method, cooperation method and sharing method are all attribute data which can be classified. Among them, preservation methods can be divided into living and frozen; cooperation methods can be divided into cooperation, independence and outsourcing; sharing methods can be divided into paid and free. These essential attribute data are added to the original three-dimensional multi-layer hierarchy classification system, and a new classification system with three-dimensional multi-layer hierarchy framework and essential attribute data is obtained. It should be noted that the previous three-dimensional multi-layer hierarchy framework is a hierarchical structure, with major, intermediate and minor categories if necessary. The forth part is the essential attribute data, in which the three classifications are independent of each other, and they are parallel. From the perspective of improving the retrieval efficiency, this is the most efficient classification method for animal model resources.

### Classification principle

The classification and coding of animal model resource data mainly involves the following principles according to its characteristics as follows. (1) Uniqueness: the categories and essential attributes of animal model resources are diversified. This makes it necessary to set a unique code for each category and attribute. One code can only represent one object in the database. (2) Regularity: the 3 dimensions and hierarchical structure design is used to make the classification and coding standard for complex resources as it is easy for coders to encode and for users to understand the code. (3) Integrity: in data classification and coding, the general content and actual needs of unique indicators of resource data are considered as a combined entity, which ensures the integrity of generality and uniqueness of data. (4) Scalability: with the new additions to the animal model resource, it is important to continuously provide new classification codes for the new resource data and to reserve appropriate backup capacity for the classification codes at each layer to allow for the continuous expansion of new resources and their descriptive data items [37].

### Classification basis and criteria

This research refers to the Science and Technology Resource Identification (GB/T 32843–2016), the *Replication Methodology of Animal Models for Human Disease*, the Classification and Coding Rules for Health Information Data Set (WS/T 306–2009) [38], the Generic Description Specification for Experimental Material Resources in *The Generic Description Specification for Natural Science and Technology Resources* [39], and the Scientific Data Management Measures (000014349/2018-00052) [40]. According to the three dimensions of system disease, animal species, and modeling methods, the hierarchical structure method is used together with essential attribute data to classify and encode the animal model resource data.

### Data characteristics

According to the first dimension of classification—the human disease [1], animal models can be classified into the following major categories: (1) cardiovascular system models, (2) digestive system models, (3) respiratory system models, (4) urinary system models, (5) reproductive system models, (6) endocrine diseases models, (7)ophthalmology and otolaryngology models, (8) oral diseases models, (9) bone diseases models, (10) skin diseases models, (11) nervous system models, (12) blood system models, (13) infectious diseases models, (14) tumor models, (15) traditional Chinese medicine (TCM) viscera dialectics models, and (16) animal models for other diseases. These categories can be further subdivided into 118 intermediate categories, which can further be subcategorized into more minor categories. In terms of the second dimension of classification—animal species, the categories include animal models based on various laboratory animals, such as mice, rats, guinea pigs, hamsters, voles, experimental rabbits, dogs, miniature pigs, and nonhuman primates. The third dimension of classification depends on the modeling method. Animal models can be divided into spontaneous, induced, and genetically engineered animal models.

The data-based expression of animal model resources involves recording, saving, and sharing data while objectively describing the essential attributes of animal model resources developed using the aforementioned methods. It also involves the process of refining these essential attributes. In this process, a large number of data items that describe the essential attributes of model resources are generated, such as model phenotype data, judgment indicators, preservation methods, and literature data. Therefore, formulating the corresponding data-saving specification and standard is the important premise and condition for the data-based expression of animal model resource and ensuring the collection, storage, and sharing of resource data [41].

Based on animal model resource data classification, hierarchical structuring is used for data coding to ensure clear hierarchy among data categories. The hierarchical structure method, also known as the line classification method, categorizes the objects to be classified into several layers according to the classification basis, arranges them into upper and lower layers, and finally assigns corresponding codes to the hierarchical categories. The classified and coded data includes the resource data of all animal models categories and their subcategory models, as well as their corresponding essential attribute data items to describe the model. On the basis of the difference nature of data, the classification code field is mainly divided into two parts: the former field which is the 3 dimensions multi-layer part is the trunk classification code, whereas the latter which is the essential attribute part is the branch classification code. Further, regular symbols that are easily recognized and processed by humans and machines are used for relevant codes to establish the classification system.

## Results and discussion

### Classification structure design

The classification of animal model resource data is categorized into three dimensions, namely, system disease, animal species, and modeling method. The classification of system disease follows the logic of the chapter arrangement of the *Replication Methodology of Animal Models for Human Disease*, such as cardiovascular, digestive, respiratory systems, etc [1].Subsequently, these major diseases are deconstructed layer by layer. Finally, the three-layer tree hierarchy comprising16 major categories, 118 intermediate categories, and more minor categories under the general category of system diseases is determined, as presented in the left part of Table 1. The classification of animal species used in the modeling process of animal model resources is divided by referring to the “Experimental Material–Laboratory Animals” in the Hierarchical Classification and Coding Table of Natural Science and Technology Resource [39] in *The Generic Description Specification for Natural Science and Technology Resources*. It comprises intermediate categories of animal species, such as mouse, rat, and guinea pig, and minor categories of animal strain, as presented in the middle part of Table 1. According to the modeling method, the animal model resources are divided into three categories, namely, spontaneous, induced, and genetic engineering, which are further divided into several subcategories. This is presented in the right part of Table 1.

**Table 1:** Classification of animal models based on 3 dimensions.

Classification of Information based on System Diseases*			Classification of Information based on Animal Species#			Classification of Information based on Modeling Method*	
Major category	Intermediate category	Minor category	Major category	Intermediate category (Animal species)	Minor category (Animal strain)	Category	Subcategory
Cardio-vascular system	Myocarditis	Coxsackie virus B3 infection myocarditis	Laboratory animal	Mouse	BALB/c mouse	Spontaneous animal model	Naturally occurring
		Cytomegalovirus infection myocarditis			KM mouse		Gene mutation
		diphtheria toxic myocarditis			C57 mouse	Induced animal model	Physical method
		…… ……			…… ……		Chemical method
	Pericarditis			Rat	SD rat		Biological method
	Hypertension				Wistar rat		…… ……
	…… ……				…… ……	Genetic engineering animal model	Transgenosis
Digestive system	Esophageal diseases			Guinea pig			Gene knockout
	Gastric diseases			Dog			Gene knock-in
	Intestinal diseases			Nonhuman primate			
					
Endocrine system	Diabetes						
	Thyroid						
	…… ……						
…… ……							
					

Source: 1) *Replication Methodology of Animal Models for Human Disease; 2) #The Generic Description Specification for Natural Science and Technology Resources; 3) *Replication Methodology of Animal Models for Human Disease

**Fig 1:**
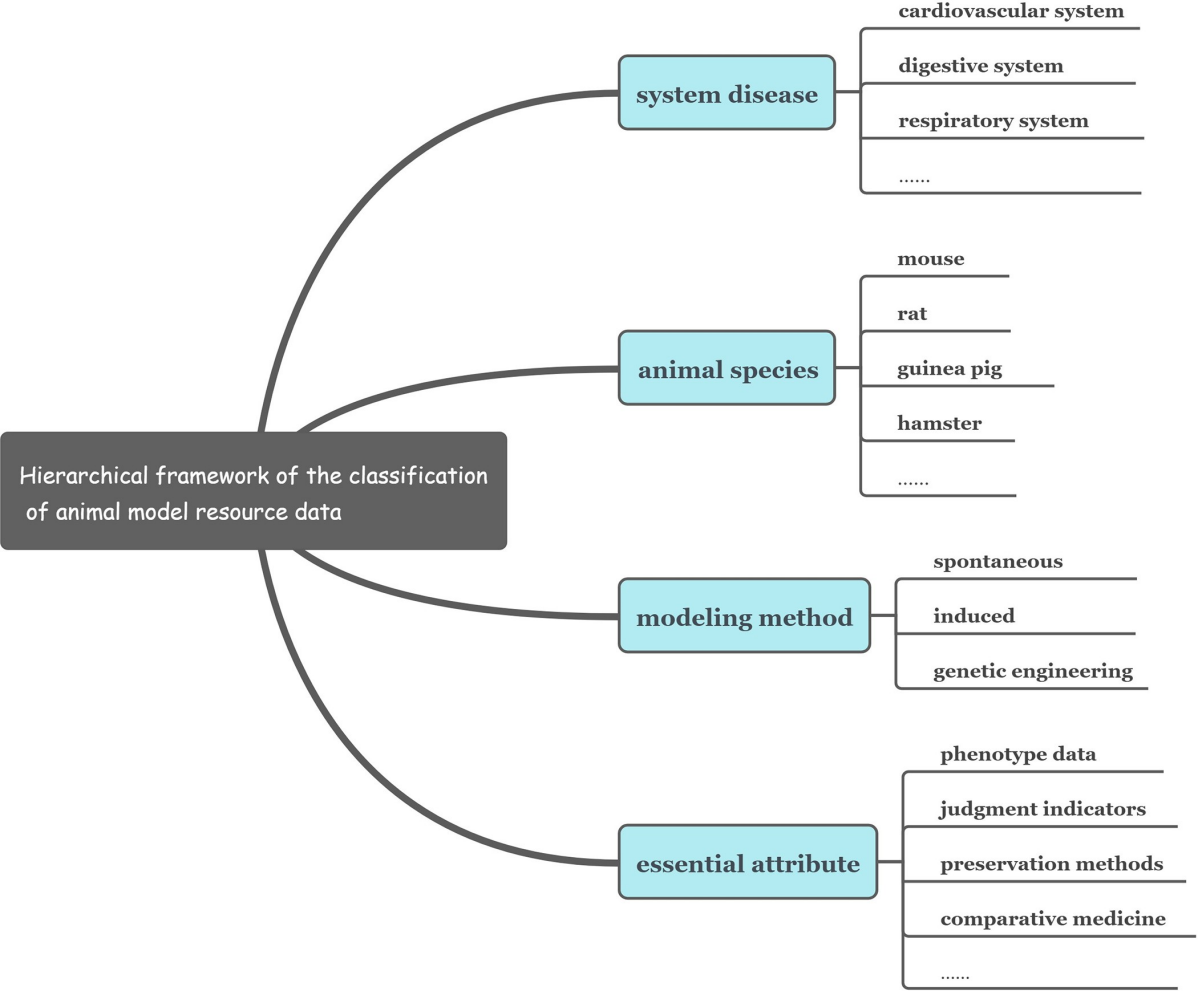
Hierarchical framework of the classification of animal model resource data.

The corresponding coding framework is set according to the classification of animal model resource data. To ensure that the coding system is hierarchical, concise, easy to operate, and in line with the various specifications and standards, the code of animal model resource data is divided into five parts, each of which corresponds to five data items, namely, identification code, system disease, animal species, modeling method, and essential attributes. Here, the second, third, and fourth parts of the code involve the classification results of the three dimensions, which are the trunk-classification codes. The fifth part of the code can be expanded into subdatasets, so it is the branch-classification code. It describes the preservation method, cooperation method, sharing method, and other essential attributes of the resources. The hierarchical framework of the data classification is presented in Fig 1.

### Specific coding methods

The classification coding of the animal model resource data comprises five modules—identification code, system disease data code, animal species data code, modeling method data code, and essential attribute data code.

According to the classification of animal model resource data, the data of system disease, animal species, and modeling method and essential attribute data are spread out and coded. The classification code is matched with the classification system, and the letter + number code is assigned.

**Fig 2:**
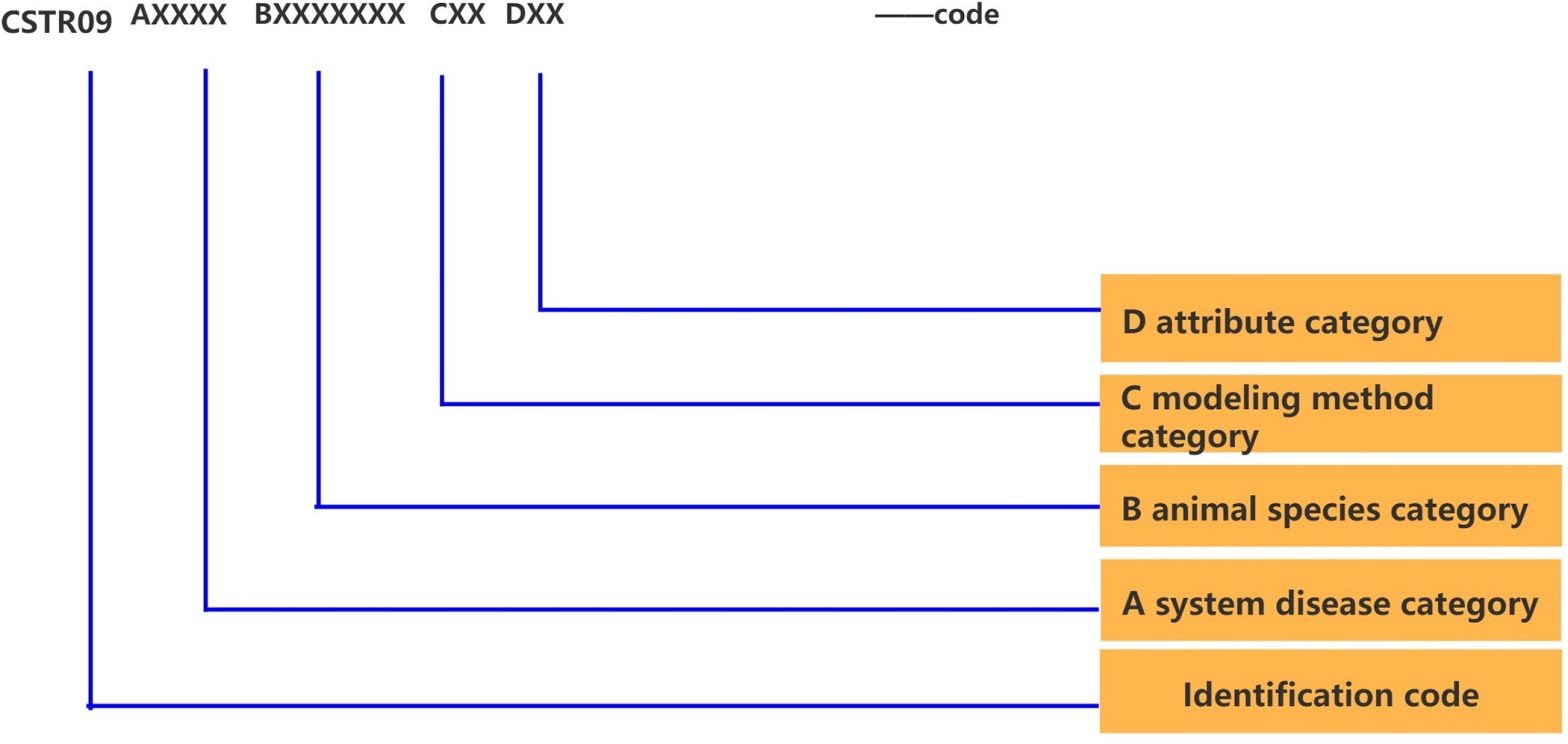
Code structure and setting.

The code of each animal model adopts a mix of English letters and Arabic numerals, and the value range of each code field location will be determined. The identification code is set according to Science and Technology Resource Identification (GB/T 32843–2016). This code is to facilitate the compatibility of this database with the general database of the China Science and Technology Resource Sharing Network in the future. The three dimensions (system disease, animal species, modeling method) and essential attribute are represented by letters A–D, respectively. Further, on the basis of this structure, the corresponding major, intermediate, and minor categories are compiled layer by layer with sequence code, and the sequence code here is generated in the dynamic way of incremental number to develop a letter + number classification code system. For example, under the classification data of system diseases, the major category corresponds to 16 major categories of system diseases, with the cardiovascular category being one of them. Similarly, the intermediate and minor categories are coded in the same way. If any layer is empty, 00 is taken as the supplementary code. It should be noted that although the codes are arranged from left to right using characters (letter + number), the codes in the three dimensions multi-layer classification structure represent a hierarchical relationship, while the classification codes of essential attributes are parallel. Finally, a complete code is combined with various categories of animal model resource classification data through this set of classification and coding method. The code structure and setting are presented in Fig 2. Subsequently, a scientific data management standard for sharing of animal model resources may be established.

According to the Chinese national standard in Science and Technology Resource Identification (GB/T 32843–2016), “The unified code of all scientific and technological resources in China is represented by CSTR [18], which is the English acronym of Chinese Scientific and Technological Resources.” In the terms of the type of scientific and technological resources, “experimental materials” refers to “Material objects with natural resource attributes, artificially cultivated or manufactured, with certain external representation and internal quality, which support and meet the needs of human scientific and technological activities.” In the Hierarchical Classification and Coding Table of Natural Science and Technology Resource, the code of “experimental material” is 09 [18]. Therefore, the animal model resource discussed in this study belongs to “experimental materials.”According to the principle of unification on the identification of scientific and technological resources, the identification code of CSTR09 is uniformly given to animal model resource.

The system disease dimension is represented by letter A. The classification data of system diseases are in the ascending order: cardiovascular system diseases, digestive system diseases, respiratory system diseases, urinary system diseases, reproductive system diseases, endocrine diseases, ophthalmology and otolaryngology diseases, oral diseases, bone diseases, skin diseases, nervous system diseases, blood system diseases, infectious diseases, tumors, TCM viscera dialectics diseases, and other diseases; these 16 major categories of human diseases are coded. In the intermediate category, take cardiovascular diseases as an example, they include myocarditis, pericarditis, myocardial infarction, arrhythmia, myocardial ischemia, arterial thrombosis, hypertension, atherosclerosis, and other items. And data are coded in this order. For the minor category, take myocarditis as an example, they also include Coxsackie virus infection, mouse cytomegalovirus infection, diphtheria toxin-induced myocarditis, and other items. And the data are coded in this order. The major, intermediate, and minor categories are coded in Arabic numbers, wherein the code length is present in the form of two digits as 00–99 (See the first part inTable 2).

**Table 2:** Data coding structure of animal model.

Data coding structure of system disease			Data coding structure of animal species				Data Coding Structure of Modeling Method			Data coding Structure of Essential Attributes		
A System disease category		Full code	B Animal species category			Full code	C Modeling method category		Full code	D Essential attribute category		Full code
Major category	Intermediate category		Major category	Inter-mediate category	Minor category		Category	Code		Attribute data item	Code	
01 cardio-vascular disease ……	01 myocarditis 02 cardio pericarditis 03 myocardial infarction ……	A0101 A0102 A0103 ……	11 laboratory animal ……	11mouse 13 rat 15 pig	001 BALB/c 002 KM 003 C57 ……	B1111001 B1111002 B1111003 ……	Spontaneous animal model Induced animal model Genetic engineering animal model	01 02 03	C01 C02 C03	Cooperation Method Sharing Method Preservation method	1/2/3 1/2 1/2	D100 D110 D111

Note: 1) During research for this paper, the classification coding of system disease is mainly coded in the major and intermediate categories. In the practical application, the minor categories can be added to the code on the basis of the major and intermediate categories. The expansion of hierarchical classification coding is conducive to improving the efficiency of the retrieval.

Note: 2) During research for this paper, the classification code of modeling method is mainly coded in three major categories. In practical application, the subcategories can be added to the code according to right part of Table 1 of this paper based on the major categories. Expansion of the hierarchical classification code is conducive to improving the efficiency of retrieval.

Animal species is represented by letter B, and it refers to the laboratory animal species in the animal model resources. The classification data coding of laboratory animal species follows the coding rules of laboratory animals in the Hierarchical Classification and Coding Table of Natural Science and Technology Resource in *The Generic Description Specification for Natural Science and Technology Resources*. This rule describes the classification code for laboratory animals. In the major category, number 11 represents laboratory animals. This code is to facilitate the compatibility of this database with the general database of the China Science and Technology Resource Sharing Network in the future. In the intermediate category, for example, number 11 represents mouse; 13 represents rats; 15represents guinea pigs; and so on [42]. In this study, based on the above rules, animal species is coded layer by layer. From the major category to the intermediate category, to the minor category, all of which are coded in Arabic numbers (See the second part of Table 2).

The modeling method is represented by letter C. It is divided into three categories, namely, spontaneous, induced, and genetically engineered animal models. The classification codes are expressed as two digit Arabic numbers and are compiled in 00–99 (See the third part of Table 2).

The essential attribute data category is represented by letter D. In addition to the trunk-classification data of system disease, animal species, and modeling method, the animal model resource also generates a large amount of essential attribute data that objectively describes the essential characteristics of the model in the animal model development process. These essential attribute data, if can be classified, are classified as branch-classification data and are encoded on their different attributes, such as cooperation method, sharing method, preservation method, etc. The cooperation method is listed in the first character position after the letter D. 1 represents independence, 2 represents cooperation, and 3 represents outsourcing. The sharing method is listed in the second character position after the letter D. 1 means paid, and 2 means free. The preservation method is listed in the third character position after the letter D. 1 means live and 2 means frozen. Essential attribute categories are coded as one-digit Arabic numbers and are compiled as 000–322 (See the fourth part of Table 2). If the data for any attribute are missing, 0 is taken as the supplementary code instead.

The classification and coding of animal model resource data comprises the overall classification and coding structure of identification code + system disease category data code + animal species category data code + modeling method category data code + essential attribute data code. Furthermore, it comprises code columns from left to right through the trunk-classification codes (including the major, intermediate, and minor category codes) and branch-classification codes, which are essential attribute category codes. Finally, a complete set of classification and coding system of animal model resource data is compiled and established. This classification and coding system follows the national identification rules of Scientific and Technological Resources as well as the generic description specifications of laboratory animal resource in the industry to enable the collection, saving, and management of general and unique data of animal model resources. It lays the foundation for the maximizing the animal model resources sharing in China. The code structure can also be expanded or reduced according to the actual situation. An example of the code structure is presented in Table 3.

**Table 3:** Code structure.

Science and technology resource identification code: CSTR09								
A System diseases		B Animal species			C Modeling method	D Attribute item	Model	Complete code
Major category	Intermediate category	Major category	Intermediate category	Minor category	Category	Data item		
01 ……	01 ……	11 ……	11 ……	001 ……	01 ……	01 ……	Cardiovascular disease Myocarditis Laboratory animal Mouse BALB/c mouse Spontaneous model Cooperation method	CSTR09: A0101B1111001C01D200

Through the data classification, the corresponding coding system is designed and coded, and the classification and coding table of a large number of animal models resource data is established. Because the creation of a complete data classification and coding table is time-consuming, this paper uses a condensed template—the classification and coding table of cardiovascular disease animal models resource data (Table 4). The models listed in Table 4 are part of the cardiovascular system models, the first of the 16 major category animal models of human diseases.

**Table 4:** Model classification and coding table (condensed).

Model code	Model classification
CSTR09: A0101B1111001C01D122	Cardiovascular disease—myocarditis—laboratory animal mouse—BALB/c mouse—spontaneous modeling—independent—free—frozen
CSTR09: A0101B1111001C01D312	Cardiovascular disease—myocarditis—laboratory animal mouse—BALB/c mouse—spontaneous modeling—outsourcing—paid—frozen
CSTR09: A0102B1127001C01D121	Cardiovascular disease—cardiopericarditis—laboratory animal—rabbit—New Zealand rabbit—spontaneous modeling—independent—free—live
CSTR09: A0102B1127001C01D322	Cardiovascular disease—cardiopericarditis—laboratory animal—rabbit—New Zealand rabbit—spontaneous modeling—outsourcing—free—frozen
CSTR09: A0103B1111001C02D121	Cardiovascular disease—myocardial infarction—laboratory animal—mouse—BALB/c mouse—induced modeling—independent—free—live
CSTR09: A0103B1111001C02D311	Cardiovascular disease—myocardial infarction—laboratory animal—mouse—BALB/c mouse—induced modeling—outsourcing—paid—live
……	……

After the results of classification and coding of animal model resources are obtained, the first thing is to measure which is more efficient between the retrieval method of this study (Hereinafter referred to as the New Method.) and the retrieval method used in the National Human Disease Animal Model Resource database (Hereinafter referred to as the Old Method.). We use an example to test it. For example, the database user wants to retrieve the model used earlier in this research whose identifier is CSTR: 16397.09.0C01001234. The classification and essential attributes of the model are as follows: model name: SARS-CoV-2 infected transgenic mice model, animal species: ICR-Tg (hACE2) transgenic mice, disease type: SARS-CoV-2 (although this is the name of the virus and the name of the disease is COVID-19, the former is used as the name of the disease in the database.), modeling method: induced method, and it also has the attributes of cooperative research and living preservation. If the classification coding method of this study is used for this model, the model code is A0312B1111017C02D201.

The first method is to use the Old Method provided by the original database. If basic search is used, we cannot get the identifier of the model. We can only type the keyword SARS in the dialog box. The search result is 18 animal models about SARS or SARS-CoV-2. Because it is impossible to refine the search results, the target model can be finally obtained through visual search among the 18 animal models. If advanced search is used, we can type SARS in the dialog box of model name field, and the other 5 fields cannot be used any more for lack of information. After retrieval, the same results are listed as the basic search.

The second method is the New Method. User adopts the model code A0312B1111017C02D201 of this study. In the above search results from Old Method, a comprehensive and in-depth review of the 18 animal models is conducted and the result shows that only one model adopts ICR-Tg (hACE2) transgenic mouse. Therefore, the B1111017 part in our code ensures that our model can be retrieved at one time.

The above experimental result shows that the New Method greatly improves the retrieval efficiency of the Old Method. A lot of repeated retrieval experiments have verified the same results.

The following is the SWOT comparison of the two methods.

#### •Purpose

The establishment of China National Human Disease Animal Model Resource database itself is a government action. The coding purpose of the original database is very clear. As a part of the China Science and Technology Resource Sharing Network, it must be completely compatible with the whole network. The coding rule must be consistent with the rules of the whole network. Due to the wide variety of resources in the big network, the resource classification and coding system must be highly comprehensive and have a certain administrative concern. The Old Method is highly consistent with the general coding rules of the whole network at the cost of sacrificing the inherent structure and specific content of this research field. Its strengths are compatibility and consistency. The New Method is only partially considered to be consistent with the overall coding rules of the China Science and Technology Resource Sharing Network, and it highlights the specific research characteristics of this research field. The strengths of the New Method are academic and professional.

#### •Efficiency

The Old Method is unlikely to retrieve target model at one time unless the specific and accurate model identifier is known. The New Method can ensure that the target model can be retrieved at one time in most cases. This strength stems from its design purpose. Its coding method fully reflects the characteristics of animal models, so the retrieval efficiency is very high.

#### •Relevance

The vast majority of researchers are doing relevance retrieval when searching in databases. They searched for relevant and enlightening materials for their research. The Old Method is convenient to search for a specific target model only when the exact model name and identifier are known accurately. The Old Method lacks the function of related expanding search on the target model. The New Method can easily obtain a batch of relevant research results by modifying one or several classification data and attribute data in the model code. The New Method is very helpful to the research. The New Methods can provide inspiration for research so it is helpful.

#### •Convenience

The New Method has the strength of which it can be mastered by the researchers only through simple training. When researchers read a code, they immediately understand what model it represents. Conversely, researchers can easily encode any model themselves by the rule. This is very convenient in the usage process and easy to be widely used.

#### •Transparency

The New Method is not only easy to understand by users but also greatly improves the retrieval efficiency, which greatly improves the accessibility of resources in the database. This makes the data more transparent. From the perspective of future development, the New Method has development opportunities. The database with government background has various functions. First is data collection and storage; second is data sharing and supporting scientific research. The Old Method fully implements the collection and storage functions through compatibility into the national resource pool, and needs to meet the data security requirements that may be specified by the government.

#### •Openness

The New Method attracts users through a more user-friendly coding system. In the long run, it will be more and more accepted by the industry, and the databases supported by it will be more and more open. This is the opportunity of the New Method and the challenge of the Old Method in the future.

#### •Compatibility

The Old Method is more compatible with government resource pool and can be supported by the government. As the government pays more and more attention in this industry, the development of the Old Method has opportunities and it is a challenge to the New Method.

#### •Industrialization

If the New Method wants to get its comparative advantage in the future, it needs to be actively industrialized. It must be generally accepted and recognized through larger scale application in the industry, and obtain financial support from the industry. This is an opportunity for the New Methods.

#### •Internationalization

At the same time, from the perspective of internationalization, because the New Method is subject to the scientific nature of this discipline, it comprises easy to understand, easy to use, user-friendly and other characteristics, it will be fully considered and evaluated for reference in the future development of international taxonomic criteria related to this kind of databases. Going global is an important opportunity for the New Method in the future.

At present, the international academic community has not formed a mainstream and widely accepted method for the classification and coding standard of the comprehensive animal model database. According to the literature collection of this paper, the construction of human disease animal model database in the field of laboratory animal research is still in its infancy. There are few national-level comprehensive animal model resource databases such as China’s National Human Disease Animal Model Resource Center database.

This makes the research in this paper unable to compare with the retrieval methods of international similar databases. This is an inherent limitation of this study. However, from the perspective of discipline development, the research in this paper provides a pioneering exploration for the future construction and development of international relevant databases, which is of scientific significance.

## Conclusion

There are some advantages of this new classification and coding system. It follows the framework of Science and Technology Resource Identification (GB/T 32843–2016); therefore, it can be embedded into a larger database within this framework and is expected to be compatible. Furthermore, it directly encodes the trunk- classification and attributes with strong coding regularity. If the code of a specific model is unknown, it can be directly deduced using the basic knowledge of classification and attributes. Based on the coding regularity, the coder can easily encode according to the objective information of the model, and the user can easily deduce the code of a specific model and retrieve it. In this set of codes, both the classification and attribute codes are added to the model code. The code simultaneously reflects multiple characteristics of the models; thus, most models can be retrieved at a time, which significantly improves the efficiency of retrieval.

Compared with other existing classification code systems proposed in these disciplines, the classification and coding system proposed in this study considers the characteristics of animal model resource data as the starting point and the retrieval needs of scientific and technological workers for animal model resource data in scientific research as the guidance. In addition, it designs and encodes on the basis of the three dimensions of system disease, animal species, and modeling method, plus the essential attribute data, thus exhibiting strong practicability. With the development and expansion of animal model resources, in practical application, the classification and corresponding code of data at all levels can be flexibly expanded, for example, by adding a new minor category under the intermediate category of the major category of system disease and adding a new attribute data item under the essential attribute dataset. The addition of the dimension of data classification and coding is conducive to improving the convenience of data integration. It also helps improve the efficiency of data sharing and retrieval.

In addition to the above mentioned limitation that this study cannot be compared with similar international studies, another limitation of this study is the representativeness of sample data to the population. China’s animal model resource database is still in the early stage of construction. Many domestic models and a few international models are collected currently in this database. This makes our sample distribution deviate from the population from the regional perspective.

In the era of big data, data classification and coding standardization is the basis for realizing data expression, storage, exchange, and integration. Moreover, it is an important method to solve the bottleneck of inconsistent data format in information construction. Particularly, data classification and coding standardization should not be ignored in database construction. Following the principles of the Scientific Data Management Measures (000014349/2018-00052), the Classification and Coding Rules for Health Information Data Set (WS/T 306–2009), this study investigated a classification and coding system for animal model resource data to provide an important basis for promoting the opening and sharing of scientific and technological resources to the society and strengthening the standardization of data description and data expression of animal model resource in China. It also provides important technical support for the construction and data-sharing of animal model resource database.

## References

[1] Zhou G.X., Gao C., Xu P., Yao M., Xie J.J., Hu J.H. 2008. Replication Methodology of Animal Models for Human Disease. Shanghai: Shanghai Science and Technology Literature Press

[2] Xia X.Z., Qin C., Qian J. 2016. Research Report on Science, Technology and Industrial Development Strategy of Laboratory Animals. Beijing: Science Press

[3] Ye Y.J., Bao X.H., Wang R.D. 2017. The Laboratory Animal Resource Survey and Development Trend in China. Beijing: Science Press. p. 169–175

[4] Núñez-Delgado A., Bontempi E., Coccia M., Kumar M., Farkas K., Domingo J. L. 2021. SARS-CoV-2 and other pathogenic microorganisms in the environment, Environmental Research. 201. n. 111606. 10.1016/j.envres.2021.111606 PMC8459334 34181924

[5] Coccia M. 2021. Technological Innovation. The Blackwell Encyclopedia of Sociology. Edited by Ritzer George and Rojek Chris. John Wiley & Sons, Ltd. doi: 10.1002/9781405165518.wbeost011.pub2

[6] Coccia M. 2017. Sources of technological innovation: Radical and incremental innovation problem-driven to support competitive advantage of firms. Technology Analysis & Strategic Management. 29. n. 9. pp. 1048–1061. 10.1080/09537325.2016.1268682

[7] Coccia M. 2022. Preparedness of countries to face COVID-19 pandemic crisis: Strategic positioning and underlying structural factors to support strategies of prevention of pandemic threats. Environmental Research. 203. n. 111678. 10.1016/j.envres.2021.111678 PMC8284056 34280421

[8] Benati I., Coccia M. 2022. Global analysis of timely COVID-19 vaccinations: Improving governance to reinforce response policies for pandemic crises. International Journal of Health Governance. 10.1108/IJHG-07-2021-0072

[9] Coccia M. 2021e. Pandemic Prevention: Lessons from COVID-19. Encyclopedia. 1. n. 2. pp. 433–444. doi: 10.3390/encyclopedia1020036

[10] Luo J.-Q., Zhao L.-Y., Yao C.-C., (…), Sun J., Li Z. 2021. Single-cell RNA sequencing reveals the spatiotemporal expression profile of SARS-CoV-2 related receptor in human and mouse testes. Journal of Shanghai Jiaotong University (Medical Science). 41(4). pp. 421–426

[11] Wang K., Han P., Huang L., (…), Wang H., Kang Y.J. 2022. An Improved Monkey Model of Myocardial Ischemic Infarction for Cardiovascular Drug Development. Cardiovascular Toxicology. 22(9). pp. 787–801 doi: 10.1007/s12012-022-09754-6 35739384

[12] Correia-Caeiro C., Burrows A., Wilson D.A., Abdelrahman A., Miyabe-Nishiwaki T. 2022. CalliFACS: The common marmoset Facial Action Coding System. PLoS ONE 17(5 May).e0266442 doi: 10.1371/journal.pone.0266442 35580128 PMC9113598

[13] Fan X., Wang X., Jiang M., Pei Z., Qiao S. 2021. An improved stacked autoencoder for metabolomic data classification. Computational Intelligence and Neuroscience. 2021.1051172 34434226 10.1155/2021/1051172 PMC8382558

[14] Li J., Tian X. 2022. Research on Embedded Multifunctional Data Mining Technology Based on Granular Computing. Computational Intelligence and Neuroscience. 2022.4825079 doi: 10.1155/2022/4825079 35769268 PMC9236840

[15] Nam B., Kim J.Y., Kim I.Y., Cho B.H. 2022. Selective Prediction With Long Short-term Memory Using Unit-Wise Batch Standardization for Time Series Health Data Sets: Algorithm Development and Validation. JMIR Medical Informatics. 10(3). e30587 doi: 10.2196/30587 35289753 PMC8965672

[16] Rodrigues J., Ziebell P., Müller M., Hewig J. 2022. Standardizing continuous data classifications in a virtual Tmaze using two-layer feedforward networks. Scientific Reports. 12(1). 12879 doi: 10.1038/s41598-022-17013-5 35896573 PMC9329455

[17] Lapchak P.A., Zhang J.H. 2018. Data Standardization and Quality Management. Translational Stroke Research. 9(1). pp. 4–8 doi: 10.1007/s12975-017-0531-9 28283966

[18] Science and technology resource identification. 2016. GB/T 32843–2016. Beijing.

[19] Bawden D. 1990. Information-systems and databases as alternatives. Altern Lab Anim. 18. pp. 83–89

[20] Bottrill K. 2004. Search Strategies on the Internet: General and Specific. Altern Lab Anim. 32(1B Suppl.). pp. 585–589 doi: 10.1177/026119290403201s96 23581141

[21] Hart L.A., Wood M.W., Weng H.Y. 2005. Effective searching of the scientific literature for alternatives: search grids for appropriate databases. Anim Welfare. 14. pp. 287–289

[22] Vadlamudi L., Jones L.A., Homayouni R. 2011. ColonyTrak: a web tool and database system for managing experimental animal models. BMC Bioinformatics. 12(7 Suppl.). A11

[23] Begley D.A., Krupke D.M., Neuhauser S.B., Richardson J.E., Schofield P.N., Bult C.J., et al. 2014. Identifying mouse models for skin cancer using the Mouse Tumor Biology Database. ExpDermatol. 23. pp. 761–763 doi: 10.1111/exd.12512 25040013 PMC4183210

[24] Morrissey B, Holen I, Chelala C, Carter P, Jones L, Blyth K, et al. 2016. Introducing SEARCHBreast: a virtual resource to facilitate sharing of surplus animal material developed for breast cancer research. npj Breast Cancer. 2. pp.16020 doi: 10.1038/npjbcancer.2016.20 28721381 PMC5515334

[25] Gunes A, Ozturk Y, Babanli A. 2009. An in vivo database model for pharmacological and physiological dosage for experimental animals.ComputBiol Med. 39. pp.590–594 10.1016/j.compbiomed.2009.04.001 19423091

[26] Lee J, Gurbuz A, Liu B.A 2010 FEB 17–18. Study-Centric Database Model for Organizing Multimodality Images and Metadata in Animal Imaging Research Facilities. Proceedings of the Medical Imaging 2010: Advanced PACS-based Imaging Informatics and Therapeutic Applications. San Diego, CA

[27] Kumar A, Wadhawan R, Swanwick C.C., Kollu R, Basu S.N., Banerjee-Basu S. 2011. Animal model integration to AutDB, a genetic database for autism. BMC Med Genomics. 4. p.15 doi: 10.1186/1755-8794-4-15 21272355 PMC3042898

[28] Todd T, Dunn N, Xiang Z.S., He Y.Q. 2016. Vaxar: A Web-Based Database of Laboratory Animal Responses to Vaccinations and Its Application in the Meta-Analysis of Different Animal Responses to Tuberculosis Vaccinations. Comparative Med. 66. pp. 119–128 27053566 PMC4825961

[29] Zanivan S, Cora D, Caselle M, Bussolino F. 2008. VRG: A database of vascular dysfunctions related genes. Comput Math Appl. 55. pp. 1068–1073

[30] Jiang X.Y., Li P.Y., Kadin J, Blake J.A., Ringwald M, Shatkay H. 2020. Integrating image caption information into biomedical document classification in support of biocuration. Database-Oxford. baaa024 doi: 10.1093/database/baaa024 32294192 PMC7159034

[31] Kong Q, Qin C. 2010. Analysis of Current Laboratory Animal Science Policies and Administration in China. ILAR J. 51. pp.E1–E10 10.1093/ilar.51.1.e1 20075493

[32] Kong Q, Qin C. 2010. Laboratory Animal Science in China: Current Status and Potential for the Adoption of Three R Alternatives. Altern Lab Anim. 38. pp. 53–69 20377304 10.1177/026119291003800107

[33] Li A.M., Zhang J.Y., Zhou Z.Y., Wang L, Liu Y.J., Liu Y.J. 2015. ALDB: A Domestic-Animal Long Noncoding RNA Database. PLoS One. 10. e0124003 doi: 10.1371/journal.pone.0124003 25853886 PMC4390226

[34] National Human Disease Animal Model Resources Center [Internet]. C2020 - [cited 2022 April 22]. Beijing (P. R. China): Institute of Laboratory Animals Science, CAMS. Available from:http://namr.org.cn

[35] Basic principles and methods for information classifying and coding. 2002. GB/T 7027–2002. Beijing

[36] Information security, cybersecurity and privacy protection—Information security controls, 5.12 Classification of information. 2022. ISO/IEC 27002:2022. Geneva

[37] Zhou T, Wang H.W., Zeng C, Zhao Y.J. 2021. RPocket: an intuitive database of RNA pocket topology information with RNA-ligand data resources. BMC Bioinform. 22. pp.428 doi: 10.1186/s12859-021-04349-4 34496744 PMC8424408

[38] The Classification and Coding Rules for Health Information Data Set. 2009. WS/T 306–2009. Beijing

[39] Cao Y.H., Liu X. 2006. The Generic Description Specification for Natural Science and Technology Resources. Beijing: China Science and Technology Press. pp. 365–366

[40] The Scientific Data Management Measures. 2018. 000014349/2018-00052. Beijing

[41] He Z.M., Xing R.C., Yue B.F., Huang R, Xue C, Wang X.M., et al. 2008. Standardization and data sharing of data expression of biological characteristics of laboratory animals. Laboratory Animal and Comparative Medicine. 28. pp.43–48. Chinese

[42] Li H.P., Wang X.M., Liu W.C., Chen M.L., Huang R. 2013. Classification and coding of biological characteristic data of laboratory animals. Acta Lab AnimSci Sin. 21. pp.52–56. Chinese

